# Micro-Arc Oxidation of Ti-45Nb Alloy: Surface Characteristics and Corrosion Behavior for Potential Biomedical Application

**DOI:** 10.1021/acsomega.5c04735

**Published:** 2025-11-12

**Authors:** Emanoel C. S. Barboza, Mayesk A. Rocha, Dario F. Rocha, Alana S. Oliveira, Matheus M. S. Reis, Sandro Griza

**Affiliations:** † PostGraduate Program in Materials Science and Engineering, 425924 Federal University of Sergipe, Av. Marechal Rondon, s/n - Jd. Rosa Elze, São Cristóvão, Sergipe 49100-000, Brazil; ‡ Department of Chemical Engineering, Federal University of Sergipe, Av. Marechal Rondon, s/n - Jd. Rosa Elze, São Cristovão, Sergipe 49100-000, Brazil

## Abstract

New β titanium alloys such as Ti-45Nb have been investigated for biomedical applications due to their promising mechanical and biocompatible properties. However, studies on micro-arc oxidation applied to Ti-45Nb surfaces are still scarce. This study explores the effect of micro-arc oxidation treatments on Ti-45Nb alloy surfaces using two different acidic electrolytes for anodization. The resulting surfaces were characterized by scanning electron microscopy, X-ray diffraction, contact angle measurements, surface energy analysis, roughness parameters, open-circuit potential, potentiodynamic polarization, and electrochemical impedance spectroscopy. Anodization significantly increased surface roughness, especially with sulfuric acid affecting the Ra parameter and phosphoric acid influencing Rt. Wettability and surface energy improved noticeably with reduced dispersive and increased polar components. The combined acid treatment (H_3_PO_4_ + H_2_SO_4_) provided superior corrosion resistance, as demonstrated by more stable Open-Circuit Potential, lower corrosion current densities, and higher impedance values. Equivalent circuit modeling suggested a more protective and complex oxide layer, attributed to greater surface heterogeneity and oxide thickness. These results indicate that anodization with the mixed acid solution is the most effective method to enhance corrosion resistance and surface performance of Ti-45Nb alloys for biomedical implants.

## Graphical abstract

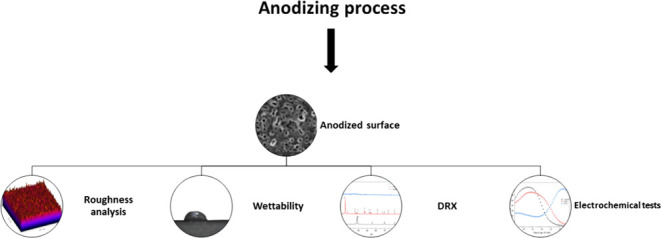

## 1 Introduction

Titanium alloys are widely recognized for their outstanding properties, including low density, excellent fatigue strength, high corrosion resistance, and biocompatibility. These attributes make them ideal for critical applications in aerospace, marine engineering, chemical processing, and, notably, biomedicine.^1–3^ Among these alloys, Ti-6Al-4V is the most commonly used for biomedical implants due to its favorable mechanical properties, as specified by the ASTM standard F136. However, the presence of aluminum (Al) and vanadium (V) in its composition raises concerns due to their potential cytotoxicity and allergenic effects in humans.^4–6^

In addition to toxicity issues, a key challenge is to reduce the elastic modulus of metallic implants to better match that of the cortical bone. To address these concerns, recent research has focused on developing new titanium alloys that eliminate toxic elements such as Al and V.

Titanium alloys can be classified into α, near-α, α + β, metastable β, and stable β phases, depending on their composition and processing.^7^ Among these, β-type titanium alloys stabilized with elements like tantalum (Ta), molybdenum (Mo), and niobium (Nb) are of particular interest due to their lower elastic modulus, which enhances biomechanical compatibility.^8–11^

Niobium has been extensively studied as a biocompatible substitute for Al and V. Ti–Nb alloys exhibit improved mechanical properties and corrosion resistance compared to commercially pure titanium, while showing no evidence of toxicity or allergic reactions.^12,13^

Alloys with 10–50 at. % Nb typically undergo a significantly reduced elastic modulus, making them suitable for load-bearing biomedical devices.^14^ Moreover, their microstructure and properties can be tailored through processing and Nb content, often surpassing conventional implant materials.^15,16^

The microstructure and grain configuration critically influence the mechanical performance of the titanium alloys. Additionally, the formation of surface oxides, particularly titanium dioxide (TiO_2_), enhances corrosion resistance and biocompatibility.^17,18^

Surface modification techniques, such as anodic micro-arc oxidation (MAO)—also known as plasma electrolytic oxidation (PEO)—have been extensively studied to further improve these surface properties. MAO produces porous oxide coatings that can be functionalized with bioactive elements like calcium (Ca), phosphorus (P), magnesium (Mg), and strontium (Sr), promoting osseointegration.^18–21^

MAO is an electrochemical technique that promotes thick, adherent oxide layers without compromising the substrate’s structural integrity.^22^ The quality of MAO coatings depends on several parameters, including applied voltage, electrolyte composition, temperature, alloy composition, and treatment duration.^7,23,24^ These factors make MAO a promising surface engineering method for enhancing the performance of titanium alloys in biomedical applications.

In this context, this study aims to investigate the influence of MAO treatment on the corrosion behavior of a Ti-45Nb alloy cold-rolled up to 0.9 strain. The coatings were evaluated in terms of pore morphology, roughness, wettability, surface energy, and corrosion performance using different electrolytes. Microscopic characterization, open-circuit potential (OCP), electrochemical impedance spectroscopy (EIS), and potentiodynamic polarization were performed in a simulated body fluid (SBF). The findings contribute to understanding the surface modifications induced by MAO and their impact on corrosion behavior, offering insights into optimizing titanium alloys for biomedical applications.

## 2 Materials and Methods

### 2.1 Sample Preparation

Discs (12.7 mm diameter, 20 mm length) were precisely cut from a commercially available Ti-45Nb alloy bar (Shaanxi OMD New Materials Sci-Tech), cold-rolled to a strain of 0.9, using a bow saw to ensure dimensional accuracy and minimize surface deformation. The specimens were then cold-mounted in a polyester resin matrix to expose one flat and polished surface suitable for metallographic and other surface analyses. Prior to mounting, the samples were thoroughly cleaned with ultrasonic agitation in ethanol and dried to remove contaminants.

All experimental procedures, including sample preparation and subsequent analyses, were carried out in triplicate to guarantee the reproducibility and statistical validity of the results. Furthermore, strict laboratory protocols were followed to minimize environmental contamination and ensure consistency across sample batches.

### 2.2 Anodization

Two electrolyte solutions were prepared for the anodization process: (i) 1.5 M sulfuric acid (H_2_SO_4_) and (ii) a mixed solution containing 1.5 M H_2_SO_4_ and 0.5 M phosphoric acid (H_3_PO_4_).^25–27^ The solutions were freshly prepared using analytical grade reagents and distilled water to ensure reproducibility and minimize contamination.

Triplicates were anodized for each test condition for 10 min in an individual 250 mL beaker containing the respective electrolyte, ensuring identical work volume and constant agitation, to maintain the homogeneity of the ionic species. Anodizing was performed using a DC power supply operated in potentiostatic mode at a constant voltage of 200 V, a condition chosen based on preliminary tests.

All anodizing procedures were performed at room temperature (25 ± 2 °C) and under controlled conditions. Upon completion of anodizing, samples were immediately removed from the electrolyte, rinsed thoroughly with distilled water to remove any residual ions or reaction products, and dried in cold air.

### 2.3 Microstructural Analysis

Longitudinal sections of the raw and anodized samples were prepared by embedding the specimens in Bakelite to ensure proper handling during surface preparation. The embedded samples were ground progressively by SiC abrasive papers up to 1200 grit to remove surface irregularities and achieve a uniform finish.

Subsequently, the specimens were polished to a mirror-like surface using diamond paste down to a 0.05 μm particle size to reveal microstructural details in high resolution. Chemical etching was then performed using a solution composed of 25 mL of H_2_O, 2.5 mL of HNO_3_, 1 mL of HF, and 7.5 mL of H_2_SO_4_ to selectively reveal the grain boundaries and phase features of the Ti-45Nb alloy.

Microstructural examinations were complemented by high-resolution surface and cross-sectional analyses using a scanning electron microscope (SEM, Vega LMS Tescan). For the SEM investigations, consistent magnification settings were maintained to enable comparative analysis among the untreated and anodized groups. The surface morphologies of the standard and treated samples were analyzed qualitatively and quantitatively, providing insights into coating homogeneity, pore distribution, thickness, and oxide layer integrity.

### 2.4 Thickness

The thickness of the coatings was determined for both anodizes. For each sample, three distinct images were acquired by microscopy, while 3 measurements were made per sample, totaling 9 measurements per condition. In each image, three line segments were drawn perpendicular to the surface by using the image scale as a reference. The values obtained were used for the statistical analysis of the average thickness of the coatings.

### 2.5 Porosity Analysis

Pore characteristics of the oxide coatings were quantitatively assessed using scanning electron microscopy (SEM) images, with three images acquired per treatment condition, in 3 different samples, to ensure representative sampling.

The measurement protocol was based on the method described by Hojat et al. (2023),^28^ adapted to the specific pore morphology of the anodized Ti-45Nb alloy. ImageJ software was employed for the quantification of the pore size and number. The image processing workflow included calibration of pixel dimensions to metric units, segmentation of pore regions, smoothing to reduce noise, verification of segmentation accuracy, and extraction of quantitative metrics such as the pore area, diameter, and pore density per surface unit.

All images were captured under consistent magnification and working distance settings to maintain comparability. The final pore data were expressed as the mean and standard deviation, ensuring traceability of the analysis.

### 2.6 Surface Roughness

Average roughness (Ra) and total roughness (Rt) were measured using a Mitutoyo SJ-410 roughness meter, with ten random measurements taken per sample to ensure representative measurements. All measurements were performed in triplicate to guarantee the reproducibility and reliability of the results.

Abbott–Firestone bearing area curves and frequency density distributions were applied to interpret the roughness profiles, providing a comprehensive understanding of the distribution of peaks and valleys across the surfaces. Additionally, surface images obtained by SEM were processed by ImageJ (converted to 8-bit format) using the waviness/roughness and plot surface plugins to visualize and quantify surface topography in both 2D and 3D.^29^

SEM-based roughness analysis complemented the contact roughness data by generating detailed topographical maps, enhancing the characterization of features such as pores and microscale protrusions. All roughness measurements were conducted in a controlled environment to minimize external vibrations and ensure the accuracy of the data.

### 2.7 Chemical Composition and X-ray Diffraction

The cross-sectional chemical composition of MAO coatings was studied using the scanning electron microscope (SEM Vega LMS Tescan) equipped with an energy dispersive X-ray spectrometer (EDS- Tescan Essence).

Crystalline phases of the alloy and oxide coatings were identified using a small-angle X-ray scattering (SAXS, Shimadzu XRD-6000) diffractometer. The measurements were performed using Cu Kα radiation (λ = 1.5406 Å) operated at 40 kV and 30 mA. Diffraction patterns were collected over the 2θ angular range from 20° to 90°, with a step size of 0.02° and a counting time of 1 s per step to ensure good resolution and signal-to-noise ratio.

Prior to measurements, the samples were cleaned with ethanol and gently air-dried to remove any surface contaminants that could interfere with the diffraction signals.

### 2.8 Wettability and Surface Energy

Contact angle measurements were performed using an optical microscope (HAIZ 1600), with 2.5 μL static droplets of distilled water and ethylene glycol applied using a precision micropipet to ensure consistent volume. Images were captured 30 s after droplet deposition—an interval selected to allow droplet stabilization on the surface, minimizing evaporation and spreading effects. Measurements were analyzed using ImageJ software with the contact angle plug-in.

Prior to each measurement, the samples were cleaned with distilled water and dried in compressed air. All tests were conducted at room temperature (25 ± 2 °C). Surface free energy was calculated using the Owens–Wendt method, which separates the total surface tension into polar and dispersive components, based on the contact angle obtained by the two probe liquids of known surface tension and polarity.^30^

1 $$\gamma_{L}\left(1+\cos\theta \right)=2{\left({\gamma_{S}}^{d}{\gamma_{L}}^{d}\right)}^{1/2}+2{\left({\gamma_{S}}^{p}{\gamma_{L}}^{p}\right)}^{1/2}$$

Where:

γ_L_: Liquid surface tension

cos *θ*_*L*_: Contact angle of liquid on solid surface

$\gamma_{s}^{d\;}and{\;\gamma }_{L}^{d}$ : Dipersive components of the surface tension of the solid and liquid,respectively

$\gamma_{s}^{p\;}and\;\gamma_{L}^{p}$ : Polar components of the surface tension of the solid and liquid,respectively

The values used for distilled water were 51.0 mN/m (polar) and 21.8 mN/m (dispersive), while for ethylene glycol, they were 30.0 mN/m (polar) and 34.0 mN/m (dispersive). All calculations were performed using the mean contact angles for each liquid, and the surface energy was reported as the average of the replicates with the corresponding standard deviation.

### 2.9 Electrochemical Tests

Corrosion behavior was evaluated through open-circuit potential (OCP), potentiodynamic polarization, and electrochemical impedance spectroscopy (EIS), using a conventional three-electrode electrochemical cell consisting of an Ag/AgCl (saturated KCl) reference electrode, a platinum counter electrode (99.99%), and the sample as the working electrode.

The tests were carried out in simulated body fluid (SBF), prepared in accordance with ISO 10993–14:2001, and maintained at 37 °C in a thermostatic bath to simulate physiological conditions. The pH was adjusted and maintained at 4.5 to replicate the acidic microenvironment typically observed during the inflammatory responses.

OCP was recorded for 3600 s to allow stabilization of the electrochemical interface, followed by EIS analysis using a 7 mV RMS sinusoidal perturbation in the frequency range from 10^5^ to 0.01 Hz. Nyquist and Bode plots were interpreted and fitted using equivalent electrical circuit models.

Subsequently, potentiodynamic polarization was performed, sweeping the potential from −1 to +1.5 V at a constant scan rate of 10 mV/s. Corrosion potential (*E*_corr_) and corrosion current density (*I*_corr_) were determined by Tafel extrapolation using the appropriate electrochemical analysis software.

All measurements were performed (MultiEmStat4 potentiostat) with rigorous cleaning of the electrodes between each test. The electrochemical curves correspond to the intermediate curves obtained from the three replications for each condition tested and the same ones used for the process of adjusting the EIS curves.

### 2.10 Statistical Analysis

All experimental data were statistically analyzed using analysis of variance (ANOVA), with the level of significance set at *p* ≤ 0.05.

## 3 Results

### 3.1 Microstructural Analysis

Figure 1 presents the microstructural and surface characteristics of the Ti-45Nb alloy. In Figure 1a, the refined microstructure of elongated beta grains is observed in the alloy after cold rolling. The etching process clearly delineates the refined and elongated grain boundaries within the beta matrix. In contrast, Figure 1b exhibits sanding marks on the surface of a representative standard sample. When compared to this standard, both anodized sample groups exhibit distinct morphological changes, stressing the effects of the anodization.

**Figure 1:**
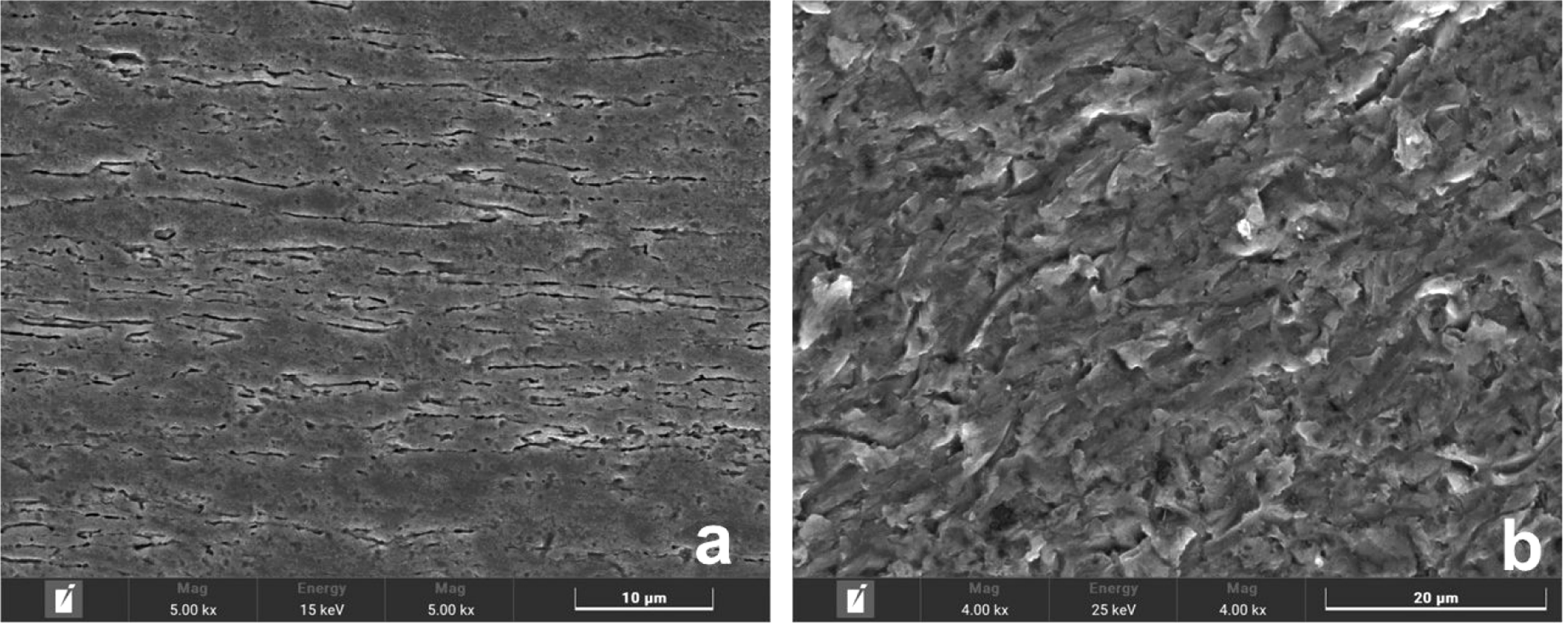
(a) Microstructure of the Ti-45Nb alloy after cold rolling with 0.9 deformation, showing refined and elongated beta grains revealed by chemical etching; (b) Micrograph of the surface of a standard sample of the Ti-45Nb alloy sanded with #600 grit, exhibiting characteristic sanding marks.

### 3.2 Surface Morphology and Coating Thickness

The samples anodized in sulfuric acid (Figure 2a) exhibited a surface morphology with a volcanic appearance, characterized by raised and irregular formations. In contrast, the group treated by the combined sulfuric acid and phosphoric acid solution (Figure 2c) showed a vermiform and irregular morphology. This irregularity can be attributed to the coalescence of adjacent pores during the anodization process, a phenomenon caused by heterogeneity in the chemical composition or in the distribution of the oxide phases formed.^31,32^

**Figure 2:**
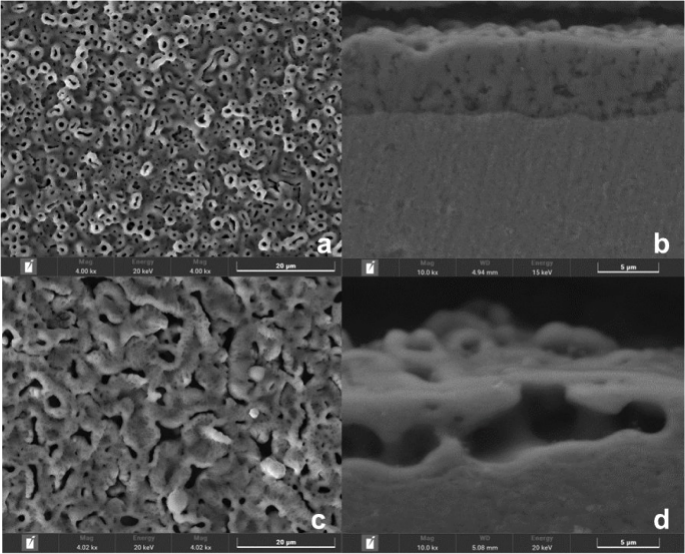
Surface morphology and coating thickness of Ti-45Nb alloy after MAO treatment: (a) surface morphology of the sample anodized in sulfuric acid showing a volcanic-like texture; (b) cross-sectional image displaying the thinner and denser coating formed in sulfuric acid; (c) surface morphology of the sample anodized in the combined sulfuric and phosphoric acid solution, exhibiting vermiform and irregular pores; (d) cross-sectional image showing the thicker coating with larger pores formed in the combined acid solution.

The thicknesses of the coatings formed by the micro-arc oxidation (MAO) process are shown in Figure 2b and d. It is observed that the coating obtained from the phosphorus-containing solution is thicker and has larger pores, whereas the coating formed only by sulfuric acid is thinner and denser. This difference can be attributed to the chemical composition of the solutions, which directly influences the formation and growth of the oxide layer during the treatment.

Quantitatively, the average thickness was 5.64 μm (standard deviation (SD) of 0.96) for the samples anodized in sulfuric acid only and 7.0 μm (SD = 1.67) for those treated with the combined acid solution. The difference was considered statistically significant (*p* = 5.92 × 10^–8^).

Despite the morphological differences, the pores on both anodized surfaces are evenly distributed. However, anodizing from sulfuric acid resulted in significantly higher pore density, with an average of 1250 pores (SD = 268), compared to 378 pores (SD = 83) for the samples treated with the combined acids. This count was performed over a fixed area of 3.9 × 10^3^ μm^2^ in both cases, and the difference was also statistically significant (*p* = 0.005).

### 3.3 Roughness

Figure 3 emphasizes the comparison of roughness between the studied groups. The surfaces anodized by sulfuric acid undergo reduction of Rt roughness when compared to that of the standard sample. The mean Rt was 2.5 μm, with the distribution ranging from 1.5 to 8 μm, where values above 2.5 μm predominated. This distribution suggests the predominance of valleys instead of peaks.

**Figure 3:**
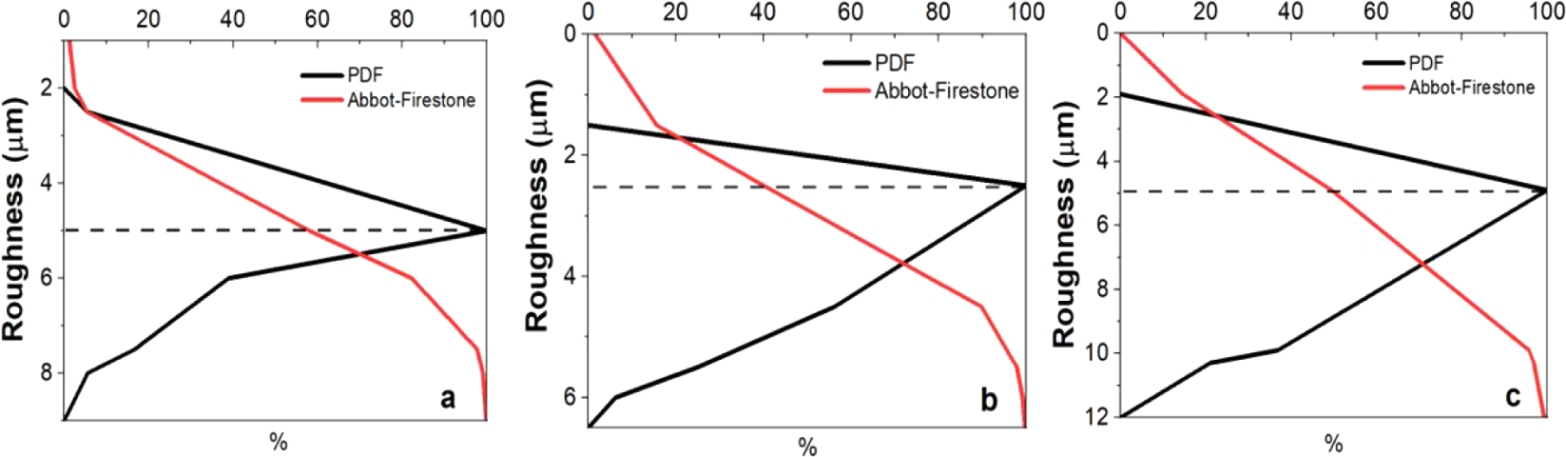
The corresponding probability density function (black line) and the Abbot–Firestone curve (red line), abscissa 0 is placed at the highest point of the surface and the axis is directed downward, within the surface: a) no treatment #600, b) H_2_SO_4_, and c) acids combined.

Figure 3a and c explain interesting differences in the density graphs as a function of the Rt. Although the midline (dashed) distributions of the standard group (sandpaper #600) and the group anodized with combined acids are close to 5 μm, the variation profiles are distinct. The standard group shows a higher frequency of values below 5 μm, indicating a surface with a predominance of peaks. In contrast, the group anodized with combined acids shows variation concentrated above 5 μm, suggesting a higher density of valleys.

Anodization induces noticeable modifications in the surface roughness of the Ti-45Nb alloy, as shown in Figure 3b. A statistically significant increase in the mean roughness of Ra and Rt was observed between the different surfaces (Ra: *p* = 0.00027; Rt: *p* = 0.00014). However, the analysis of multiple comparisons for the Ra parameter, using Tukey’s test, revealed that only the group anodized in combined acids denoted significant difference if compared to the others. The comparison between the standard group and the group treated with sulfuric acid did not show statistical significance (*p* = 0.29080).

For the Rt parameter, although ANOVA denoted significant differences between the treatments, Tukey’s test indicated that only anodizing in the sulfuric acid solution presented a difference statistically significant if compared to the other treatments. The comparison between the anodizing in the combined solution and the standard group, on the other hand, did not exhibit a significant difference (*p* = 0.90664).

Despite these topographical differences, the variations in surface density among all samples remain within a range considered to be suitable for biological applications. According to the literature, surface roughness between 20 nm and 10 μm significantly impacts the biocompatibility of materials, promoting the initial adhesion of cells and biological macromolecules.^33^

The analysis of topographic images obtained via scanning electron microscopy (SEM) using ImageJ software enables a qualitative assessment of the surface morphology across the different conditions (Figure 4). The standard samples exhibit a relatively balanced topography characterized by an even distribution of peaks and valleys. In contrast, the anodized surfaces (Figure 4b and c) present a more irregular morphology, marked by a predominance of valleys. Notably, the sulfuric acid-anodized surface (Figure 4b) denotes smoother transitions between peaks and valleys, resulting in a more uniform and lighter appearance, which contrasts with the other samples that show greater heterogeneity in surface topography.

**Figure 4:**
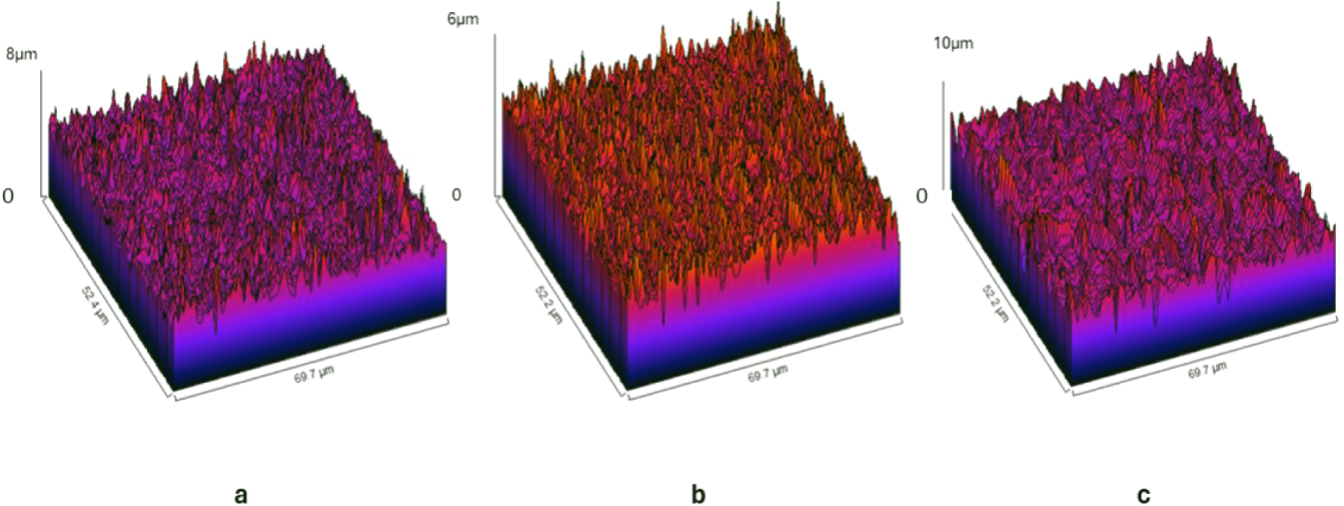
Three-dimensional modeling of Rt roughness from SEM micrographs: (a) standard samples; (b) anodized samples with sulfuric acid solution; (c) anodized samples with a combined acid solution.

In these images of Figure 4, lighter regions (orange) represent peaks, whereas darker regions (pink/purple) correspond to valleys. The standard surface demonstrates a more homogeneous distribution of the peaks and valleys. Conversely, the anodized surfaces exhibit more irregular and heterogeneous morphologies, particularly the sulfuric acid-anodized group, which shows smoother transitions and a more consistent overall appearance.

### 3.4 Chemical Composition and X-ray Diffraction

The results of the EDS analysis of the treated regions can be seen in Figure 5, corresponding to the different treatments: (a) H_2_SO_4_ and (b) H_3_PO_4_. In both samples, the formation of an oxygen-rich layer is observed. In the case of H_3_PO_4_ treatment, all of the phosphorus concentration present is located in the layer formed by the MAO process. In the sample treated with H_2_SO_4_, in addition to oxygen, Nb and Ti levels are identified, which is corroborated by XRD analysis, which indicates the presence of peaks corresponding to TiO_2_ and Nb.

**Figure 5:**
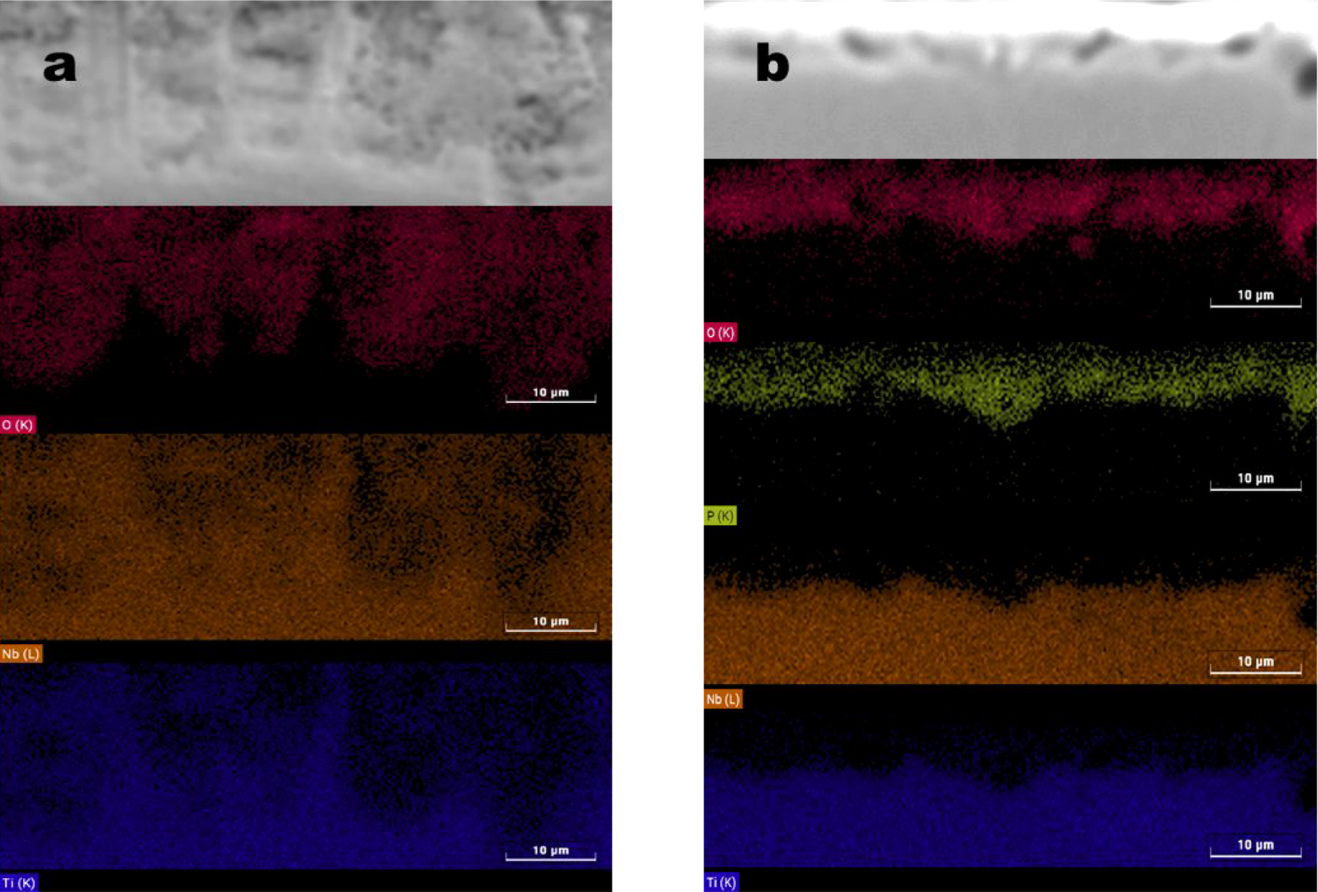
EDS elemental mapping in the cross section of the MAO process layers with solution a) sulfuric acid and b) combined acids.

The analysis explained in Table 1 reveals variations in the chemical composition as a function of the different treatments applied. Phosphorus is present only upon treatment with a solution containing phosphoric acid, although in reduced concentrations. In addition, a lower diffusion of oxygen in the alloy is observed, as indicated by the lower percentages detected.

**Table 1:** Chemical Composition in Percent of the Surface Treated with Different Acid Solutions

	**1**	**2**	**3**	Average	Standard deviation
**H**_**2**_ **SO**_**4**_					
Ti	28.4	30.78	30.37	29.85	1.27
Nb	27.72	29.52	28.97	28.73	0.92
O	43.88	39.7	40.66	41.41	2.18
**H**_**3**_ **PO**_**4**_					
Ti	38.65	33.37	35.67	35.89	2.64
Nb	28.71	24.54	26.64	26.63	2.08
O	30.1	38.19	34.37	34.22	4.04
P	2.54	3.9	3.32	3.25	0.68

Although the analysis of oxygen in EDS is imprecise, we can infer from the analysis that there is a formation of oxides and that in addition, the analysis allows the proper identification of the other elements.

The β-phase microstructure of the alloy substrate was identified by SAXS analysis (Figure 6). Similar diffraction patterns of titanium alloys with predominant β-phase structures (COD 9008554) have also been reported.^34–36^ Anodizing in the sulfuric acid solution led to the formation of titanium oxides in both rutile (COD 9004143) and anatase (COD 9008216) phases, in agreement with findings by Liao et al. (2020) and Coan et al. (2025).^37,38^ It is still possible to suggest the presence of Nb in the grazing angle analysis, based on the COD 9008546 pattern, in which peaks are observed that can be confused with the characteristics of Ti oxides and β-Ti.

**Figure 6:**
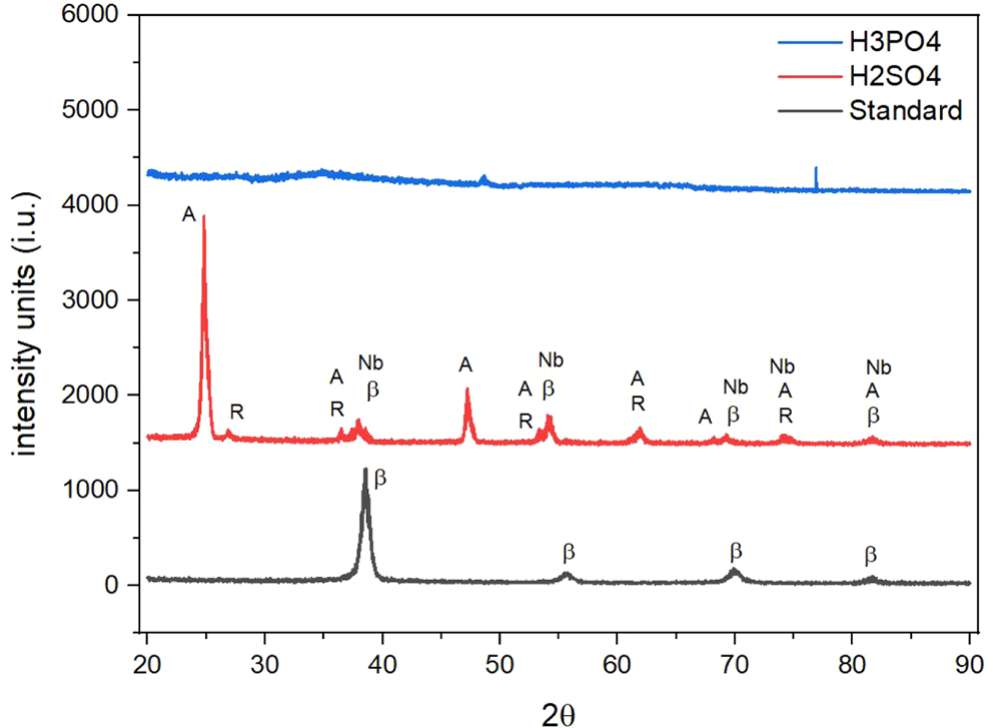
Diffractograms of the Ti-45Nb alloy where standard: surface sanded with #600 grit paper H2SO4: anodized in 1.5 M sulfuric acid solution (H_2_SO_4_) and H3PO4: anodized in a mixed solution of 1.5 M sulfuric acid and 0.5 M phosphoric acid (H_2_SO_4_+ H_3_PO_4_).

In contrast, anodizing in a mixed solution of sulfuric and phosphoric acids resulted in the formation of an amorphous surface coating, which exhibited no significant diffraction peaks—a feature commonly observed in rapid solidification and heterogeneous chemical composition systems.^35^ The absence of β-phase peaks in these groups suggests the formation of a thicker oxide coating, preventing the small-angle X-ray scattering beam from penetrating the underlying Ti-45Nb substrate.

### 3.5 Wettability and Surface Energy

Contact angle measurements with water revealed that the standard Ti-45Nb group exhibited an average contact angle of 64.6° (SD = 4.0°). Anodization in different electrolytes significantly reduced the contact angle, reaching 7.2° (SD = 12.4°) for the group treated with sulfuric acid and 43.4° (SD = 21.5°) for the group treated with the combined acid solution. This reduction indicates an increase in surface hydrophilicity, which is associated with enhanced surface bioactivity and an improved potential for biomedical applications.^39–41^

As shown in Figure 7, the untreated (standard) surface exhibited the lowest total surface energy (sum of polar and dispersive components), calculated using the Owens–Wendt method, when compared to the surfaces treated via the MAO process. Among the anodized samples, those treated solely with sulfuric acid exhibited slightly higher surface energy than those treated with an additional phosphoric acid component. However, one-way ANOVA revealed that only the group treated with sulfuric acid (SO_4_) showed a statistically significant difference compared to the standard group (*p* < 0.05; *p* = 0.00080). No statistically significant differences were observed between the acid-treated groups (*p* > 0.05; *p* = 0.13240).

**Figure 7:**
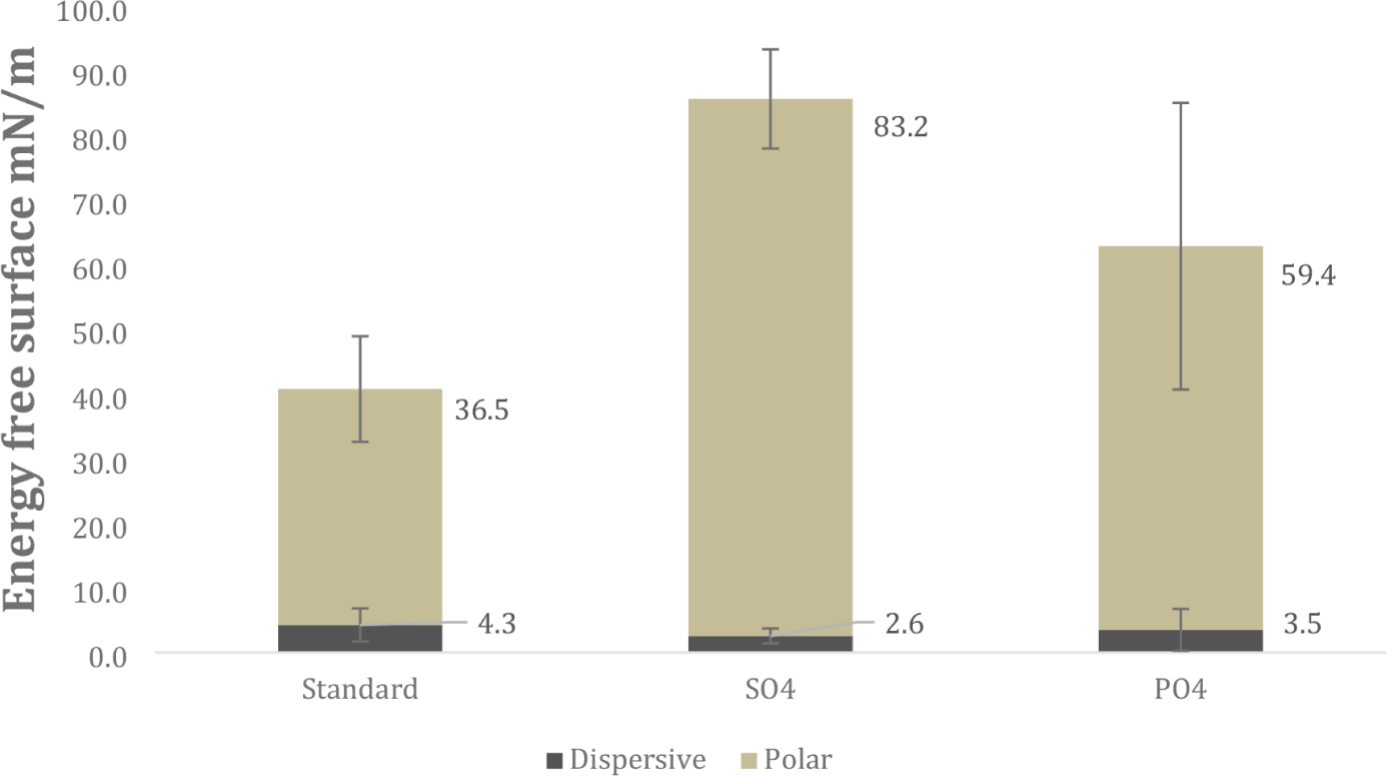
Dispersive and polar components of surface energy for the samples. The surfaces anodized by the MAO process exhibit enhanced wettability, as demonstrated by the increased polar component of the surface energy.

Consistent with the statistical results obtained for total surface energy, the ANOVA analysis for the polar component revealed a significant difference only between the standard group and the group anodized with sulfuric acid (*p* < 0.05; *p* = 0.00190).

### 3.6 Corrosion Behavior

#### 3.6.1 Open-Circuit Potential

Figure 8 exhibits the open-circuit potential (OCP) curves measured in simulated body fluid (SBF). The average OCP values in V followed the order: H_3_PO_4_ > H_2_SO_4_ > standard, indicating that higher OCP corresponds to more stable surface oxide coatings and increased corrosion resistance of the material in the tested environment.^42–44^ Among the treatments analyzed, the Ti-45Nb alloy anodized in the mixed sulfuric and phosphoric acid solution exhibited the highest OCP, suggesting that this treatment produced the most stable oxide coating.

**Figure 8:**
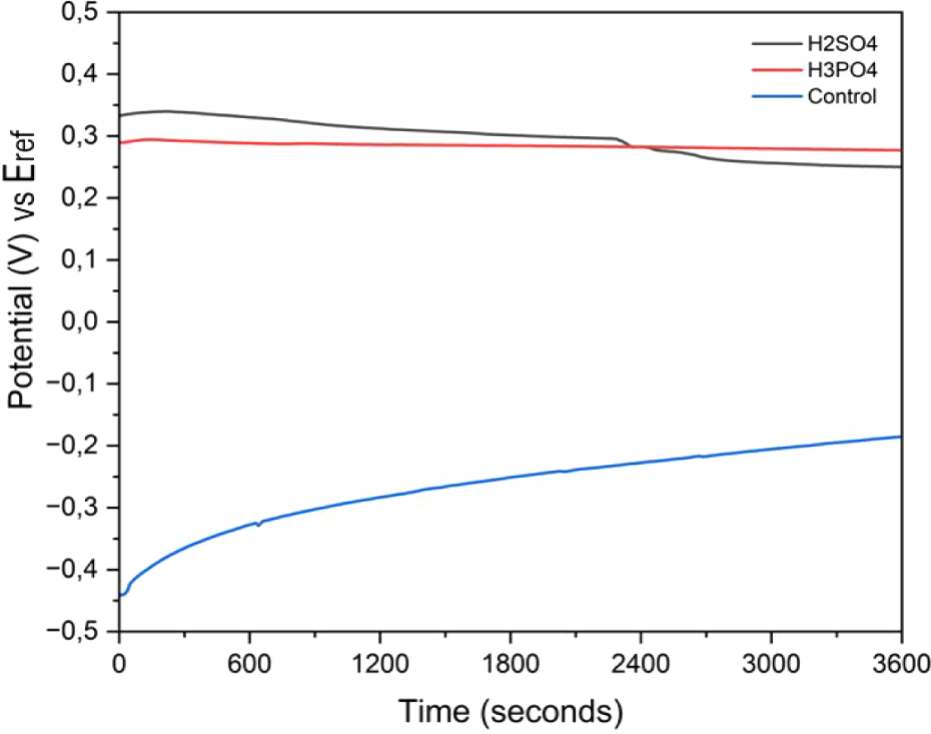
Open-circuit potential (OCP) curves of the Ti-45Nb alloy under different surface treatments: standard, anodized in sulfuric acid solution, and anodized in combined sulfuric and phosphoric acid solution.

Additionally, Figure 8 shows the progressive increase in the level of OCP over time for the untreated alloy, indicating that surface passivation occurs gradually within the observed time frame. In contrast, the anodized groups sustained stable OCP throughout the test period, which denotes the formation of denser and more uniform oxide coatings, contributing to improved surface stability.^43^ These results further indicate that oxide coatings formed via micro-arc oxidation (MAO) present superior electrochemical stability and enhanced corrosion resistance compared to untreated surfaces.

#### 3.6.2 Potenciodynamic Polarization

The electrochemical polarization tests in Figure 9 and Table 1 showed significant variations in the density of the *I*_corr_ corrosion current and in the *E*_corr_ corrosion potential after the different anodizings. A reduction in the density of the corrosion current was observed for the anodizing by combined acids, while the anodizing by sulfuric acid resulted in an increase in the density, in relation to the standard sample. The *E*_corr_ of the nonanodized samples was −0.50 V, reducing to −1.12 V after sulfuric acid anodizing. On the other hand, anodizing with phosphoric acid significantly increased the corrosion potential, reaching −0.27 V.

**Figure 9:**
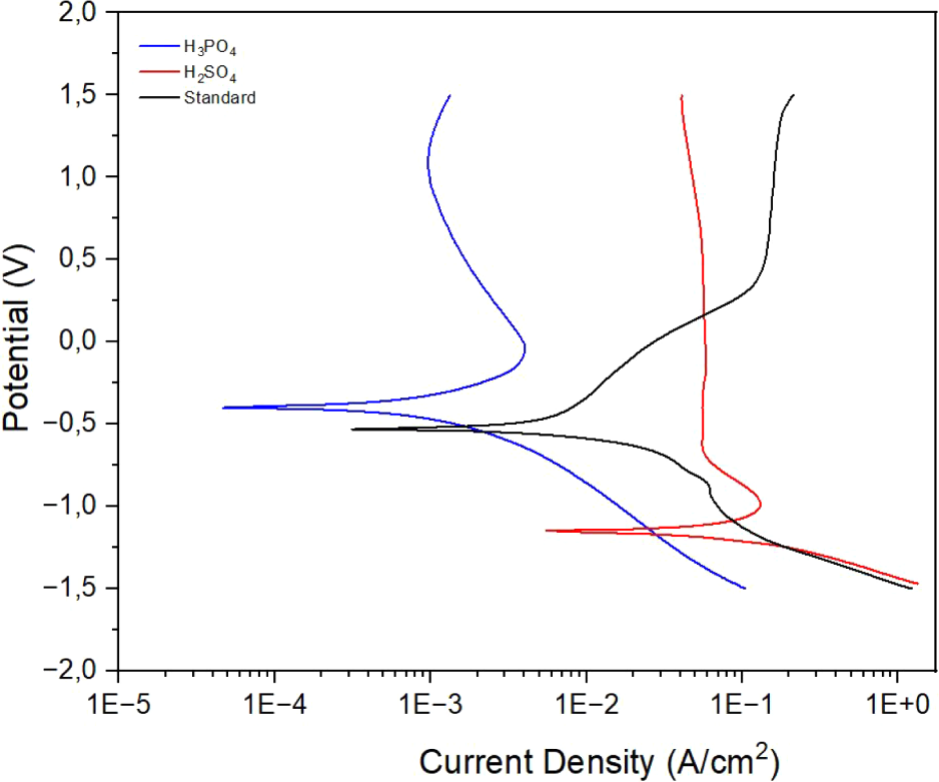
Potentiodynamic polarization curves of the Ti-45Nb alloy under different conditions: standard samples, anodized in a sulfuric acid solution, and anodized in a combined sulfuric and phosphoric acid solution.

Regarding the corrosion current density, the standard samples presented 5.4 × 10^–3^ A/cm^2^, while the samples anodized with sulfuric acid and combined acids exhibited 45.4 × 10^–3^ A/cm^2^ and 3.8 × 10^–3^ A/cm^2^, respectively. These findings indicate that the phosphoric acid treatment significantly improved corrosion performance, which is evidenced by the increase in *E*_corr_ and the reduction in current density.^45,46^

Table 2 presents the electrochemical parameters analyzed, where βa and βc correspond respectively to the slopes of the anodic and cathodic branches, *E*_corr_ represents the corrosion potential, *I*_corr_ the corrosion current density, and Rp the polarization resistance, which was given by eq 2.^45^

2 $$Rp=\beta a\beta c/\left(2,303\left(\beta a+\beta c\right)I_{corr}\right)$$

**Table 2:** Data Given from Polarization by the Taffel Method, Where βa and βc Correspond to the Slopes of the Anodic and Cathodic Branches, *E*_corr_ Represents the Corrosion Potential, *I*_corr_ the Corrosion Current Density, and Rp the Polarization Resistance

Polarization Data	*E*_corr_ (V)	*I*_corr_ (A/cm^2^)	–βa (V dec^–1^)	βc (V dec^–1^)	Average Rp (kOhm cm^2^)
standard	–0.50 (0.02)	5.4 × 10^–3^ (0.01)	6.55 (0.1)	2.24 (0.12)	134.35
H_2_SO_4_	–1.10 (0.02)	4.5 × 10^–2^ (0.008)	5.44 (2.5)	5.46 (2.47)	16.29
H_3_PO_4_	–0.27 (0.31)	4.0 × 10^–3^ (0.004)	3.68 (0.56)	2.61 (0.67)	173.83

The *E*_corr_ values indicate a thermodynamic trend favoring the group anodized with the combined acid solution, which exhibited more positive corrosion potentials. This suggests lower electrochemical reactivity and, consequently, reduced susceptibility to corrosion.^45^

In addition, the Rp results demonstrate that the samples anodized with the combined acid solution showed superior corrosion behavior compared to both the standard and samples anodized only by sulfuric acid. This behavior indicates enhanced thermodynamic and kinetic stability against the corrosive environment, thereby decreasing the likelihood of material degradation over time.^45^

#### 3.6.3 Eletrochemichal Spectroscopy Impedance

Electrochemical impedance spectroscopy (EIS) was applied to assess the corrosion behavior of the samples by analyzing both the real and imaginary components of the impedance. The corresponding Nyquist and Bode plots are presented in Figure 10, and the fitted parameters are summarized in Table 2.

**Figure 10:**
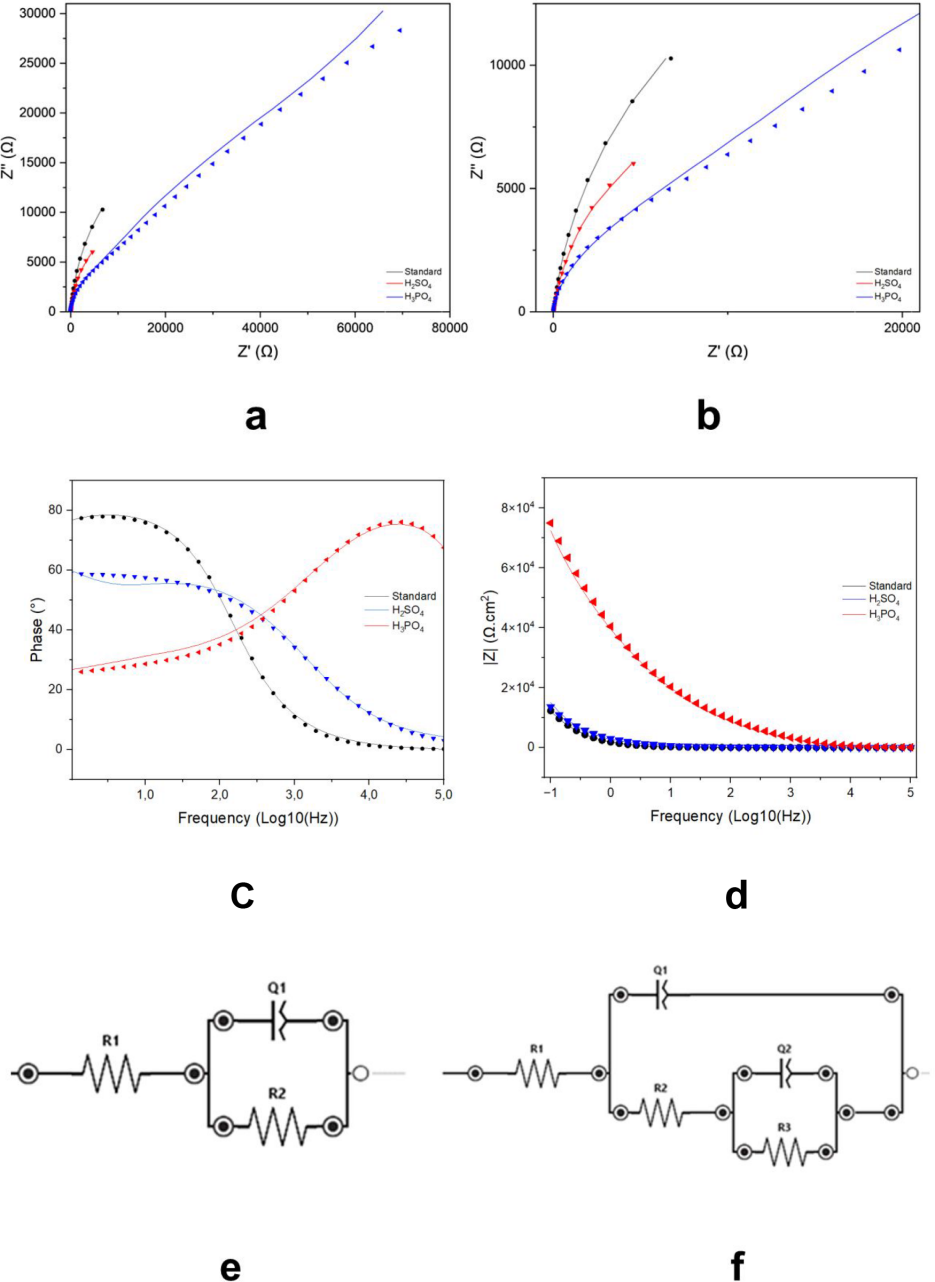
Nyquist plots (a, b), Bode plots (c, d), and equivalent electrical circuit models used for fitting the electrochemical data (e, f) of Ti-45Nb alloy surfaces under different treatments: standard, anodized in sulfuric acid solution, and anodized in combined sulfuric and phosphoric acid solution.

The MAO treatment using the combined acid solution resulted in a larger capacitive arc if compared to the untreated Ti-45Nb substrate, indicating enhanced corrosion resistance.^45,47^ As illustrated in Figure 10a and b, both the standard samples and the group anodized in sulfuric acid exhibited a single capacitive arc, which is characteristic of the formation of a passive oxide film on the surface of the Ti-45Nb alloy.

Regarding the Bode graph and the phase angle, a larger and broader phase angle suggests better corrosion resistance.^47^ Figure 10c shows that the sample anodized with the combined acid solution has the widest range in the phase angle. This feature can be linked to the superior corrosion resistance observed in this sample group.

In the Bode graph in Figure 9d, the standard and sulfuric acid-anodized samples showed the lowest impedance modulus at low frequencies. This observation indicates lower corrosion resistance when compared to the samples anodized with the combined acid solution. The impedance modulus (|*Z*|) increased after the MAO treatment. This increase confirms the protective effect of the ceramic coating formed during the process, as reported by Matykina et al. (2016)^48^ and Li et al. (2024).^49^ The impedance at a low frequency (|*Z*| at 0.01 Hz) also followed this trend. Higher values at this frequency are associated with improved corrosion resistance, as supported by literature Badr et al. (2024).^45^

The equivalent circuit shown in Figure 10e–f simulates the electrical parameters of the material. For the standard group and for the sulfuric acid anodized group, the Randles model was adequate to describe the TiO_2_ barrier and its interaction with the corrosive medium (Figure 10e). For the group anodized with combined acids, it was necessary to add a second constant-phase element (*Q*_2_) and an additional resistance (*R*_3_), representing the interaction of the solution with the pores and the inner layer of the coating. The higher corrosion performance observed in these cases may be related to the greater heterogeneity, porosity, and/or surface roughness.^45^

Table 3 shows that the Ti-45Nb substrate treated with the combined acid solution presents a significantly higher polarization resistance (Rp) compared to those of the other groups. The χ^2^ values in the same table indicate the precision of the circuit fitting; values lower than 1 suggest high fitting accuracy. These results confirm the validity of the equivalent circuit model applied.

**Table 3:** EIS Data^a^

**Data**	***R***_**1**_ **(Ohm·cm** ^**2**^ **)**	***Q***_**1**_ **(μT)**	***n***_**1**_	***R***_**2**_ **(Ohm·cm** ^**2**^ **)**	***Q***_**2**_ **(μT)**	***n***_**2**_	***R***_**3**_ **(Ohm·cm** ^**2**^ **)**	**Average χ^2^**
**Standard**	16.1	1.2 × 10^–4^	0.89	1.4 × 10^11^	-	-	-	0.15 × 10^–9^
**H_2_SO**_**4**_	18.7	1.1 × 10^–4^	0.67	7580	-	-	-	8.4 × 10^–5^
**H_3_PO**_**4**_	14.5	1 × 10^–7^	0.94	4637	0.78	0.53	5.7 × 10^14^	0.0063

a Where *R* identifies the resistances *Q*, capacitance and the χ^2^, representing the precision of the circuit fitting.

## 4 Discussion

The micro-arc oxidation (MAO) process occurs in three main stages: the initial formation of a gaseous film and bubble generation; the emerging of high-field regions associated with the presence of electrolyte anions; and, finally, the plasma discharge in oxide film defects, once the gas barrier is broken.^50^ The coatings formed typically exhibit a structure comprising a dense inner barrier and a porous outer coating, a characteristic observed in both treatments with acid solutions in the present study. This structure can enable the mechanical integration with bone tissue and enhance adhesion at the implant–bone interface.^23^

Table 1 presents the chemical composition of the sample surfaces. The alloying elements exhibit concentrations below their nominal values, indicating that the MAO treatment effectively promoted the incorporation of electrolyte-derived elements (phosphorus and oxygen) under both conditions. EDS mapping further confirms this incorporation, showing a uniform distribution of oxygen on the surface of samples treated with the sulfuric acid solution and both phosphorus and oxygen on those treated with the combined acid solution.

Coatings produced by anodization techniques, such as MAO, often result in low-crystallinity coatings, particularly when the coating is applied to metal alloys like titanium.^35^ This phenomenon is noticeable in the anodized samples with the combination of acids, in which the oxides formed are predominantly amorphous, which occurs due to the extremely rapid cooling of the oxide coating during the process.^7,51^

The plasma discharges generated during the treatment release a significant amount of localized thermal energy, while the electrolyte solution promotes the immediate cooling of the coating. Consequently, there is insufficient time for atoms to reorganize into stable crystalline structures, allowing the formation of amorphous coatings.^41–54^

Roughness parameters, such as average roughness (Ra) and total roughness (Rt), provide valuable insights into the topographic features of biomaterial surfaces. The results indicate that anodization promotes significant changes in the surface morphology of the alloy with an increase in both Ra and Rt. This change agrees with the enhanced surface irregularity exposed in the SEM micrographs Figures 2 and 3. These topographic changes are critical, as biomaterial surfaces with suitable roughness have been linked to improved interactions, particularly with respect to cell adhesion.^33,55^

The one-way ANOVA test revealed statistically significant differences between the treated samples and the standard samples, confirming the effectiveness of anodization in modifying surface topography. These changes may contribute to better interactions with the biological environment. Noticeably, the anodization with sulfuric acid exhibited a reduction of roughness, with the average Rt of 2.5 μm, which could suggest enhanced biocompatibility, as surfaces with higher valley density tend to allow cell adhesion and growth.^42^

The reduction of contact angle, the increase of polar force, and the rise of surface energy can be attributed to the high pore density formed by the MAO treatment, the increase in surface roughness, and the presence of oxygen-containing functional groups (−OH^–^ and −O^2–^) on the surface.^56^

Surface wettability directly influences cell attachment. The initial adhesion occurs through physicochemical interactions between cells, biological macromolecules, and the alloy’s surface.^57^ These interactions include ionic forces that enable protein adsorption, which is a critical step for cellular responses of the biomaterial. In general, increased wettability, which was observed with the decrease of the contact angle in the anodized samples if compared to the standard samples, it can enhance the interaction between the implant surface and the biological environment, enabling the promotion of cell adhesion.^39–41^

The pronounced porous structure of these coatings can serve as a reservoir for active biological components. The rough and porous surface morphology increases the surface area available for tissue cell attachment and reduces the time required for implant anchorage compared to nonporous surfaces.^58,59^

Micro-arc oxidation (MAO) using a combination of acids significantly enhanced the corrosion resistance of the Ti-45Nb alloys. Open-circuit potential (OCP), electrochemical polarization, and electrochemical impedance spectroscopy (EIS) analyses indicated the formation of a more stable and corrosion-resistant oxide layer. Elevated OCP values and reduced current density in the polarization curves confirmed the improved protective performance. In contrast, MAO treatment with a sulfuric acid solution produced inferior corrosion resistance, performing worse than that of the untreated.

EIS provided a complementary perspective. The group anodized with combined acids showed a higher impedance modulus and phase angle. These results emphasize the integrity and resistance of the oxide coating. The equivalent circuit model suggested a more complex and robust oxide coating for the anodized group with a combined acids. This characteristic is likely related to increased heterogeneity or surface roughness.^45^

In summary, the combined acid treatments resulted in a more stable oxide coating, offering superior corrosion protection, which is crucial for ensuring the longevity and biocompatibility of the implants.

## 5 Conclusions

This study dealt with MAO treatment on the surface of the Ti-45Nb alloy for use in implants. Two acid solutions were used for anodization, from which surface properties were analyzed with techniques such as scanning electron microscopy, chemical composition, X-ray diffraction, wettability and surface energy, roughness, open-circuit potential, polarization, and impedance spectroscopy. The results allowed the following conclusions to be raised:

- For the first time, the results of anodizing via MAO in Ti-45Nb alloys are presented from the perspective of biomedical use.
- Anodization significantly increased the roughness parameters of the alloys, with the roughness parameter Ra being statistically significant for the group treated with H_2_SO_4_, and Rt being significant for the treatment with the H_3_PO_4_ solution.
- EDS analyses show that there is a difference between the compositions of the treated surfaces and the substrate.
- Wettability was improved with the anodization of the Ti-45Nb alloy.
- The surface energy of the anodized alloy increased with the increase of the polar component.
- Surface treatments with combined phosphoric and sulfuric acids, at 200 V, for 10 min, at 220 °C, on the Ti-45Nb alloy resulted in superior corrosion behavior if compared to treatments with sulfuric acid alone or the standard group.
- The open-circuit potential (OCP) analysis indicated that the anodization with combined acids exhibited greater stability of the oxide coating.
- Electrochemical polarization tests and electrochemical impedance spectroscopy (EIS) confirmed that the anodization with the combined acid solution denoted lower corrosion current densities and higher corrosion resistance, reflecting a more stable oxide coating.
- The equivalent circuit model suggests that the oxide coating formed is more complex and resistant in the samples anodized with combined acids, likely due to increased heterogeneity, surface roughness, and chemical composition.

Based on the results obtained in the present study, it can be concluded that the most suitable anodization treatment for the Ti-45Nb alloy for implant purposes was that achieved with the combined acid solution, as it demonstrated the most favorable performance between all analyses conducted.
